# An RNAi screen of the kinome in epithelial follicle cells of the *Drosophila melanogaster* ovary reveals genes required for proper germline death and clearance

**DOI:** 10.1093/g3journal/jkaa066

**Published:** 2021-03-06

**Authors:** Diane P V Lebo, Alice Chirn, Jeffrey D Taylor, Andre Levan, Valentina Doerre Torres, Emily Agreda, Sandy B Serizier, Allison K Lord, Victoria K Jenkins, Kimberly McCall

**Affiliations:** 1 Department of Biology, Boston University, Boston, MA 02215, USA; 2 Program in Biochemistry and Molecular Biology, Boston University, Boston, MA 02215, USA; 3 Program in Molecular Biology, Cell Biology, and Biochemistry, Boston University, Boston, MA 02215, USA

**Keywords:** Drosophila, phagocytosis, kinase, cell death, phosphoinositide 3-kinase

## Abstract

Programmed cell death and cell corpse clearance are an essential part of organismal health and development. Cell corpses are often cleared away by professional phagocytes such as macrophages. However, in certain tissues, neighboring cells known as nonprofessional phagocytes can also carry out clearance functions. Here, we use the *Drosophila melanogaster* ovary to identify novel genes required for clearance by nonprofessional phagocytes. In the *Drosophila* ovary, germline cells can die at multiple time points. As death proceeds, the epithelial follicle cells act as phagocytes to facilitate the clearance of these cells. We performed an unbiased kinase screen to identify novel proteins and pathways involved in cell clearance during two death events. Of 224 genes examined, 18 demonstrated severe phenotypes during developmental death and clearance while 12 demonstrated severe phenotypes during starvation-induced cell death and clearance, representing a number of pathways not previously implicated in phagocytosis. Interestingly, it was found that several genes not only affected the clearance process in the phagocytes, but also non-autonomously affected the process by which germline cells died. This kinase screen has revealed new avenues for further exploration and investigation.

## Introduction

Every day, billions of cells die within the human body (Renehan *et al.* 2001; Arandjelovic and Ravichandran 2015; Elliott and Ravichandran 2016). These cells die by as many as a dozen different mechanisms, including apoptosis and necrosis (Kroemer *et al.* 2009; Galluzzi *et al.* 2018). Cell corpses that remain in the body have been implicated in several diseases such as cancer, autoimmunity, and neurodegenerative disorders (Thompson 1995; Fuchs and Steller 2011; Fu *et al.* 2014; Poon *et al.* 2014). To avoid this damage, dying cells and their debris are cleared away by phagocytes.

Phagocytic cells fall into two categories, professional and nonprofessional, depending on their primary function. The primary function of professional phagocytes, such as macrophages, is to clear away bacteria, dying cells and debris. Nonprofessional phagocytes, such as certain epithelial cells, have a separate primary function that is supplanted when they encounter a dying cell (Parnaik *et al.* 2000; Wood *et al.* 2000; Arandjelovic and Ravichandran 2015).

The best characterized form of phagocytosis, that of apoptotic cells, has been divided into a four-step process—chemotaxis, recognition, engulfment, and degradation. In chemotaxis, an apoptotic cell releases “find-me” signals, such as ATP, that form a concentration gradient to attract phagocytes (Elliott *et al.* 2009; Arandjelovic and Ravichandran 2015). Once a phagocyte is within range of the apoptotic cell, its engulfment receptors, *e.g.* CED-1/Drpr/Megf10, recognize and bind to “eat-me” signals, such as phosphatidylserine, exposed on the outer leaflet of the apoptotic plasma membrane (Fadok *et al.* 1992; Zhou *et al.* 2001; Lauber *et al.* 2004; Serizier and McCall 2017). Binding of “eat-me” signals and engulfment receptors leads to signaling within the phagocyte that induces cytoskeletal rearrangements to form a phagocytic cup and direct the process of engulfment (Nakaya *et al.* 2008; Arandjelovic and Ravichandran 2015; Serizier and McCall 2017). Once the apoptotic cell is engulfed into a phagosome, it undergoes corpse processing where the phagosome matures and fuses with a lysosome, thus acidifying and degrading the cell corpse (Arandjelovic and Ravichandran 2015; Meehan *et al.* 2016a; Serizier and McCall 2017).

The first studies to identify phagocytic genes were performed in *Caenorhabditiselegans* where several cell death defective (CED) genes were found to be responsible for apoptotic corpse clearance (Hedgecock *et al.* 1983; Ellis *et al.* 1991). These genes were later organized into two partially parallel phagocytic pathways: *ced-1*, *ced-6*, *ced-7* and *ced-2*, *ced-5*, *ced-12*. Each of these pathways relays the “eat me” signal to *ced-10*, a GTPase responsible for regulating the cytoskeletal rearrangements that form the phagocytic cup (Kinchen *et al.* 2005). Orthologs for each of these pathways were later identified in *Drosophila melanogaster* and mammals—*ced-1/Drpr/Megf10, ced-6/Ced-6/GULP, ced-7/Eato/ABCA1/7* and *ced-2/Crk/CrkII*, *ced-5/Mbc/Dock180*, *ced-12/Ced-12/ELMO1/2*, and *ced-10/Rac1/Rac1*(Ellis *et al.* 1991; Franc 2002; Freeman *et al.* 2003; Kinchen *et al.* 2005; Mangahas and Zhou 2005; Santoso *et al.* 2018). These two pathways, however, are just a small sample of all the genes regulating phagocytosis.

Since these pathways were elucidated, dozens of genes that regulate phagocytosis have been identified in *Drosophila* and mammals, *e.g. crq*/*CD36* and components of the Jun-N-terminal kinase (JNK) pathway (Franc *et al.* 1996; Etchegaray *et al.* 2012; Jenkins *et al.* 2013; Xiao *et al.* 2015; Hursh *et al.* 2016; Meehan *et al.* 2016b; Serizier and McCall 2017; Zheng *et al.* 2017; Yalonetskaya *et al.* 2018)*.* That being said, there are still several gaps in our knowledge, such as how the JNK pathway and *crq* interact with the core signaling pathways. In addition, we now know that cells can die in a dozen different ways and their corpses can be cleared by nonprofessional phagocytes, thus the gaps in our knowledge are actually quite vast.

**Figure 1:**
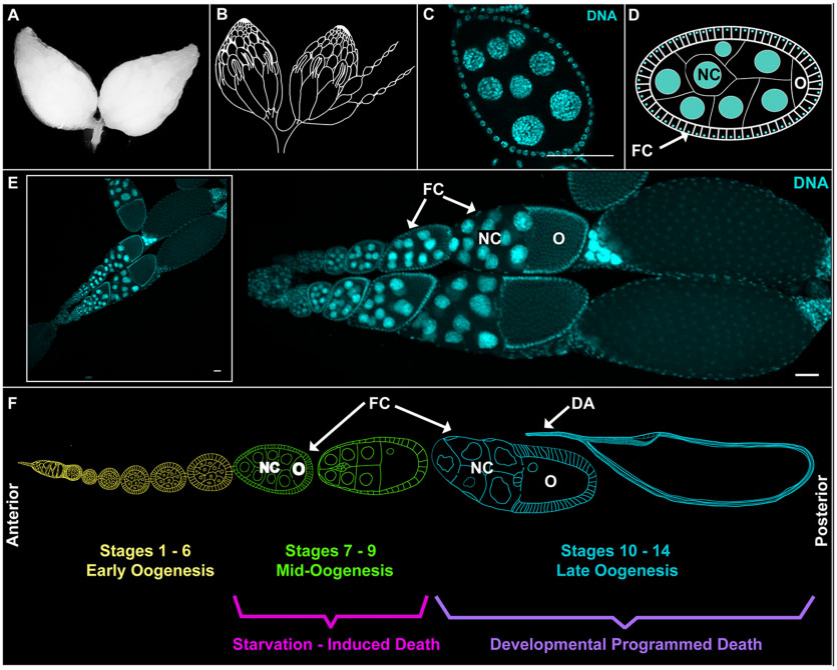
The *Drosophila* ovary is composed of developing egg chambers. (A) Two *Drosophila* ovaries. (B) Cartoon of *Drosophila* ovaries, illustrating strings of developing egg chambers called ovarioles. The drawing was adapted from Mahowald and Kambysellis (1980). (C) The central plane of a mid-stage egg chamber from a *GR1>Luc^RNAi^* (control) fly. DNA is labeled with DAPI (cyan). (D) Cartoon of mid-stage egg chamber. Each egg chamber consists of three cell types, nurse cells (NC), oocyte (O), and a surrounding epithelial FC layer. In the cartoon, the nuclei are in cyan and membranes are white. (E) Two ovarioles from a single *Drosophila* ovary. The inset is the original image from which these ovarioles were taken. (F) Cartoon of an ovariole. Early stages are labeled in yellow. Mid-oogenesis is labeled in green. Late oogenesis is labeled in cyan. Egg chambers complete the 14 stages of oogenesis while progressing from anterior to posterior in the ovariole. Death events can be induced by starvation during mid-stage oogenesis (stages 7–9) and are seen at the completion of development of healthy egg chambers (stages 12–14). The ovariole drawing was adapted from Frydman and Spradling (2001). All scale bars depict 50 μm.

The ovary of *D. melanogaster* has provided a unique advantage for studying cell death and corpse clearance by nonprofessional phagocytes as germ cells can die by several separate mechanisms during oogenesis and are removed by epithelial follicle cells (FCs) (King 1970; Buszczak and Cooley 2000; Bellen *et al.* 2011; Jenkins *et al.* 2013; Peterson *et al.* 2015). In addition, the ovary is a large tissue and its structure is highly organized and easy to visualize (Figure 1, A and B). Each ovary is composed of 15–20 strings of progressively developing egg chambers (Figure 1, C and D) called ovarioles (Figure 1, B, E, and F). Within an egg chamber are three morphologically distinct cell types—the germline-derived syncytium of polyploid nurse cells (NCs) and diploid oocyte (O), which is surrounded by a single layer of somatically derived epithelial FCs (Figure 1, C–F) (King 1970; Jenkins *et al.* 2013).

As an egg chamber progresses from the anterior to posterior of the ovary, it proceeds through 14 stages of development in which the oocyte and NCs grow in size while the FCs mature and reorganize themselves (Figure 1, E and F). Toward the end of development, during stages 10–14 (Figure 1F) the NCs transfer their cytoplasmic contents and many of their organelles through ring canals and into the oocyte in an actin-dependent process called dumping (King 1970; Spradling 1993; Jenkins *et al.* 2013). Once dumping is complete, the NCs die and are cleared by the nonprofessional phagocytes of the ovary, the FCs, which subsequently die themselves. Previous studies have found that developmental nurse cell death occurs largely independent of apoptosis and autophagy genes (Peterson and McCall 2013), and that FCs are required for complete nurse cell death (Timmons *et al.* 2016; Yalonetskaya *et al.* 2020). This form of cell non-autonomous cell death has been called phagoptosis or phagocyte-dependent cell death (Timmons *et al.* 2016; Mondragon *et al.* 2019).

FCs also clear away dying germline cells after stress-induced apoptotic death in mid-oogenesis. Before egg chambers begin the final stages of oogenesis, if conditions are poor, such as during protein starvation or chemical exposure, the entire germline undergoes regulated cell death that is predominantly apoptotic, thus avoiding the energy-consuming process of vitellogenesis (Giorgi and Deri 1976; Chao and Nagoshi 1999; Nezis *et al.* 2000; Drummond-Barbosa and Spradling 2001; McCall 2004; Pritchett *et al.* 2009; Jenkins *et al.* 2013; Serizier and McCall 2017). As death proceeds, the FCs synchronously enlarge and engulf the dying germline. Once the germline is cleared, the FCs also die (Etchegaray *et al.* 2012).

Several previous studies have focused on characterizing candidate genes and have identified over two dozen genes required in FCs for proper clearance of dying germ cells (Etchegaray *et al.* 2012; Meehan *et al.* 2015a; Timmons *et al.* 2017; Santoso *et al.* 2018; Mondragon *et al.* 2019). To identify novel pathways regulating clearance in the ovary, we conducted an unbiased screen of the *Drosophila* kinome for phagocytosis phenotypes in the ovary. As kinases are important regulatory enzymes that play a role in a variety of signaling pathways and are conserved across evolution, they provide a strong starting point for identifying a comprehensive cell corpse clearance network.

A collection of 328 RNAi fly lines representing 224 kinase genes were screened using the GAL4-UAS system. Of these 224 genes, 54 demonstrated a phenotype during developmental death and clearance while 58 demonstrated a phenotype during starvation-induced regulated cell death and clearance. Eighteen of these genes showed severe phenotypes during developmental death and clearance whereas 12 genes showed severe phenotypes during starvation-induced cell death and clearance. This cell corpse clearance kinome identifies several avenues for future study.

## Materials and methods

### Identification of kinase library

Gene Ontology (GO) terms associated with *Drosophila* genes (FlyBase2013_01) were searched for “kinase activity” and other kinase-related terms (Thurmond *et al.* 2019). A list of 359 genes was produced (Supplementary Table S1). After further research into the nature of these genes, four genes were manually excluded as they did not encode kinases.

### Fly stocks and husbandry

RNAi stocks corresponding to the kinase library (Supplementary Table S2) generated by the Transgenic RNAi Project (Perkins *et al.* 2015) were obtained from the Bloomington *Drosophila* Stock Center. Additional RNAi lines for *for*, *nemo*, and *Sdr*, were obtained from the Vienna *Drosophila* Resource Center (Dietzl *et al.* 2007). The *w^1118^* strain, *luc^RNAi^*, and age-matched siblings lacking either the driver or RNAi construct were used as controls.

**Figure 2:**
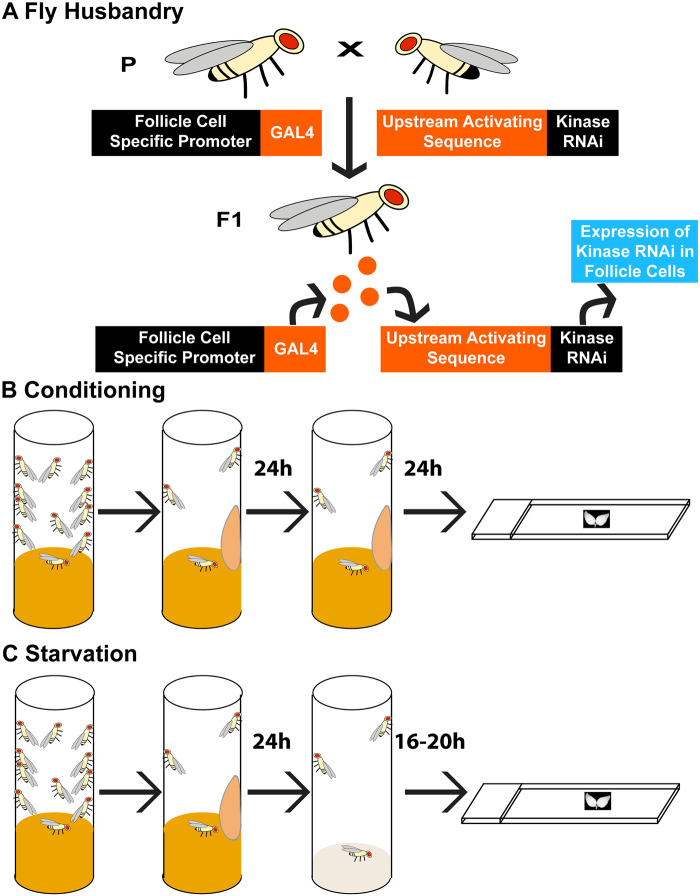
Experimental approach to detect different cell death phenotypes. (A) The bipartite Gal4-UAS system was used to express kinase RNAi constructs in the FCs of *Drosophila* ovaries. A FC specific promoter drives expression of the Gal4 transcription factor. This construct is expressed in one parental fly. An upstream activating sequence (UAS) is bound to a gene of interest in the other parental fly. When flies are mated, their progeny have Gal4 expressed in the FCs which can then bind the UAS to promote the expression of the desired gene or, in this case, kinase RNAi. (B) Experimental set up for analysis of developmental NC death. Flies with the desired genotype were conditioned with extra yeast paste which was applied to the inner wall of a vial twice over 48 h. Ovaries were then dissected, stained, and imaged. (C) Experimental set up for starvation induced cell death. Flies with the desired genotype were conditioned for 24 h with extra yeast paste. After 24 h, flies were transferred to an apple juice agar vial to starve the flies of protein. Flies were kept on apple juice agar for 16–20 h until their ovaries were dissected, stained, and imaged.

*GR1-GAL4 G89/TM6B* virgin females were crossed with males containing UAS-RNAi lines to knock down expression of kinase genes (Figure 2A). *GR1-GAL4* drives expression in the FCs of the ovary (Goentoro *et al.* 2006) while *G89 (G00089)* is a GFP gene trap that expresses endogenous GFP in the germline (Morin *et al.* 2001) and can be used to visualize engulfment (Meehan *et al.* 2015b).

Flies were raised at 25°C on standard molasses/yeast/agar/cornmeal food. To obtain well developed ovaries, flies <20 days old were conditioned on standard food plus fresh yeast paste for 48–72 h, changing fresh yeast paste every 24 h (Figure 2B). To induce cell death during mid-stage oogenesis, flies <20 days old were conditioned on standard food plus fresh yeast paste for 24–48 h, and then starved on apple juice agar for 16–20 h (Figure 2C) (Pritchett and McCall 2012; Meehan *et al.* 2015b).

### DAPI and antibody staining

Ovaries were dissected in either 1X PBS or Grace’s medium and fixed for 20 min at room temperature (RT) in 200 μL Heptane, 100 μL of 16% (wt/vol) paraformaldehyde (PFA, Electron Microscopy Sciences, open <7 days), and either 300 μL of Grace’s medium (Lonza) or 1X PBS. After rinsing twice with 1X PBT (1X PBS with 0.1% Triton X-100), tissue was washed in PBT 3X over 1 h at RT.

For antibody staining, samples were then blocked at RT for an hour in PBANG [1X PBS + 0.5% Bovine serum albumin (BSA; Fisher) + 5% normal goat serum (NGS; Life Technologies)]. Samples were then incubated in PBANG and diluted primary antibody overnight at 4°C. After rinsing 2X with PBT, samples were washed 4x for 30 min with PBT + 0.5% BSA at room temperature (total wash time 2 h). Each sample was then incubated with diluted secondary antibody in PBANG (>300 μL) for 1 h, protected from light. Again, samples were rinsed twice with 1X PBT and washed with PBT + 0.5% BSA for 2 h, changing washes at least 4 times. Samples were rinsed with 1X PBS.

Samples were incubated in 1–2 drops of Vectashield + 4ʹ,6- diamidino-2-phenylindole dihydrochloride (DAPI) (Vector Laboratories) overnight at 4°C before mounting on slides.

The primary antibody α-Discs large (Dlg) [Developmental Studies Hybridoma Bank (DSHB)] was used at 1:100 dilution in PBANG. The secondary antibody goat-α-mouse Cy3 (Jackson ImmunoResearch) was used at 1:200 dilution in PBANG.

### LysoTracker staining

After dissection, ovaries were incubated with LysoTracker Red DND-99 (Invitrogen) 1:50 in 1X PBS for 6 min while periodically flicking the tube. Samples were rinsed once with 1X PBS, left to sit in 1X PBS for 2 min, then washed for 30 min in 1X PBS with at least 2 wash changes. Next, tissue was fixed for 20 min with Grace’s Fix as described above. Fix was removed and ovary tissue was washed 3X in PBT over 15 min. After removing wash, samples were stained with DAPI and mounted as described above.

### Imaging

Samples were imaged on an Olympus BX60 upright fluorescence microscope or Olympus FV10i confocal microscope. Images were processed and compiled in Image J, Adobe Photoshop, and Adobe Illustrator.

**Figure 3:**
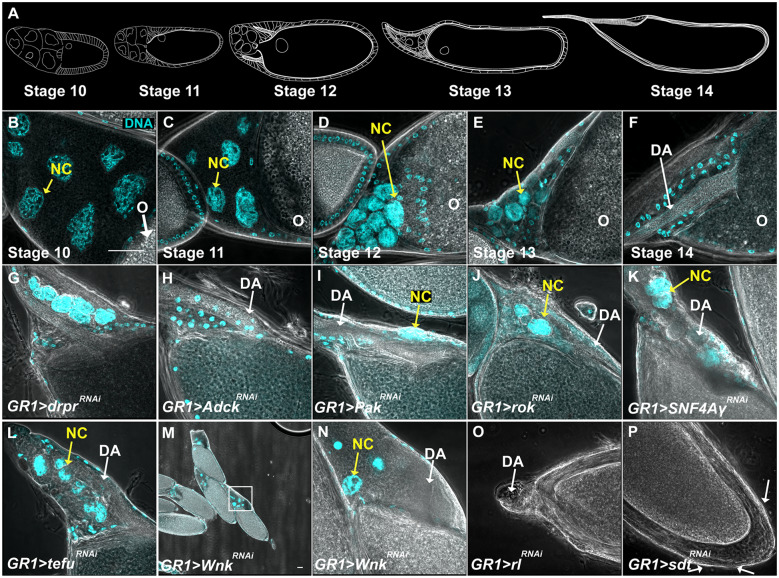
Classification of defective developmental death and clearance using the persisting nurse cell nuclei phenotype. (A) Cartoon of late stage oogenesis morphology adapted from King (1970). As egg chambers progress through the final stages of oogenesis, the volume of the NCs (anterior, left) decreases while that of the oocyte (posterior) increases. The NCs are then depleted and the oocyte development is complete with the growth of DA and a chorion shell. (B–F) Anterior (NC) region of *GR1>Luc^RNAi^* (control) egg chambers during the final stages of oogenesis. DNA is labeled with DAPI (cyan). NC nuclei are indicated by yellow arrows, and small nuclei are FCs. (B) At stage 10, NC chromatin is dispersed and the nuclei are surrounded by cytoplasm. (C) During stage 11, NCs dump their cytoplasmic contents into the oocyte (O). (D and E) By stage 12, dumping is complete and throughout stages 12 and 13, the NC nuclei are cleared. (F) By stage 14, all nuclei are cleared and the oocyte has fully developed DA. (G–O) Representative images of anterior regions of stage 14 egg chambers demonstrating different developmental death and clearance phenotypes. DNA labeled with DAPI (cyan). (G) A stage 14 egg chamber from *GR1>drpr^RNAi^* fly demonstrates a very strong PN phenotype. (H) *GR1>Adck^HMS02533^* demonstrates no PN phenotype. (I) *GR1>Pak^HM05156^* demonstrates a mild PN phenotype. (J) *GR1>rok^JF03225^* demonstrates a moderate PN phenotype. (K) *GR1>SNF4Agamma^JF02060^* demonstrates a strong PN phenotype. (L) *GR1>tefu^HMS02790^* demonstrates a very strong phenotype. (M and N) *GR1>Wnk^HMJ02087^* demonstrates a dumpless phenotype. (M) Several *GR1>Wnk^HMJ02087^* egg chambers shown at 100X. (N) A 600X image of square in box M. (O**)** *GR1>rl^HMS00173^* demonstrates a gnarled dorsal appendage phenotype. (P) *GR1>sdt^HMS00953^* demonstrates a “bumpy” chorion phenotype (white arrows). All scale bars depict 50 μm. Scale bar in B is representative of all images in this figure unless otherwise indicated.

**Figure 4:**
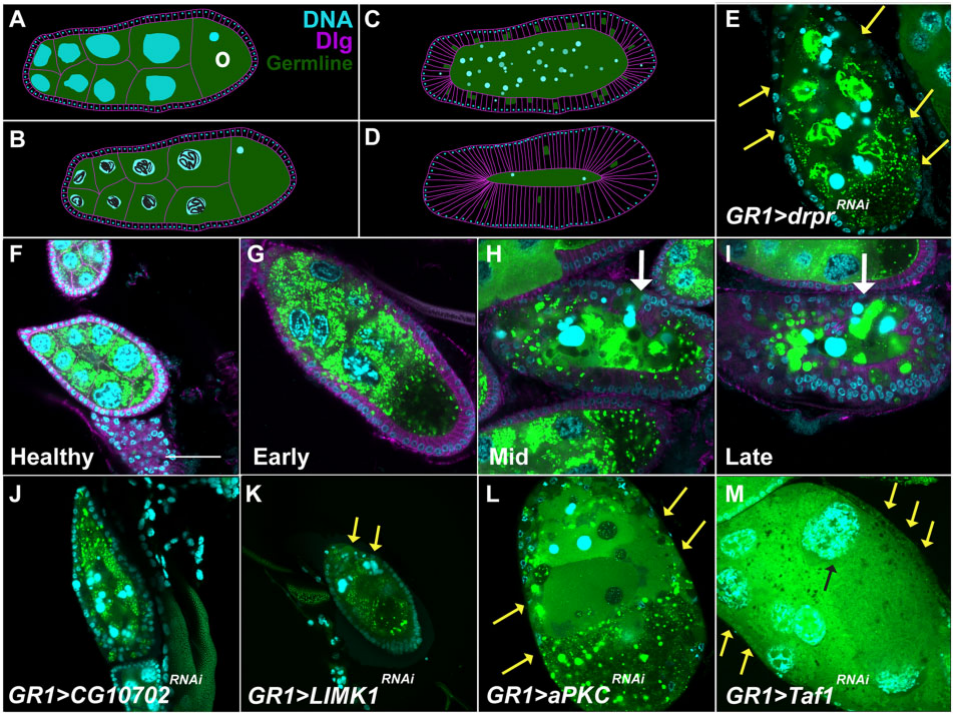
Engulfment phenotypes during starvation-induced death during mid-oogenesis. (A–D) Cartoon of starvation-induced egg chamber death. DNA is depicted by cyan, germline cytoplasm is depicted in green, and membranes are depicted in magenta. (A) A healthy, mid-oogenesis egg chamber is composed of germline cells that are approximately equal in size that contain large, dispersed, polyploid nuclei surrounded by a layer of FCs. The posterior-most germline cell is the developing oocyte (O). (B) Early phase dying egg chamber where the NC nuclei of the egg chambers become disorganized, condense, and begin to fragment. (C) Mid-phase dying egg chamber where the NC nuclei further degrade and the FCs increase in size and engulf the germline material. (D) Late phase dying egg chamber where all of the germline has been consumed. (E–M) Representative egg chamber images depicting different germline death and clearance phenotypes during mid-oogenesis. DNA labeled with DAPI (cyan), germline cytoplasm is labeled with GFP (green), and membranes are labeled with Dlg (magenta). White arrows identify the egg chamber of interest. Yellow arrows indicate missing FCs. (E) *GR1>drpr^RNAi^* egg chamber shows pronounced engulfment defects with missing FCs and unengulfed germline. (F–I) Healthy, early phase, mid- and late-phase dying egg chambers from a protein-starved *GR1>Luc^RNAi^* control fly. (J) *GR1>CG10702^HMS02499^* produces a wild-type phenotype. (K) *GR1>LIMK1^JF02063^* produces a weak clearance defect in that some of the FCs have been eliminated before the dying germline has been cleared. (L) *GR1>aPKC^JF01966^* demonstrates a strong clearance defect in that most FCs have disappeared before the dying germline has been cleared. (M) *GR1>Taf1^HMS00416^* demonstrates an “undead” phenotype where no FCs are seen surrounding the living germline. All scale bars depict 50 μm. Scale bar in (F) is representative of all images in this figure.

During late-stage oogenesis, the stage progression of egg chambers was assessed using dorsal appendages (DA). As depicted in Figure 3, A–F, stage 12 nurse cells lack cytoplasm or a dorsal appendage, stage 13 nurse cells have a thin, budding DA, and stage 14 egg chambers are characterized by fully formed DA. To visualize phases of death during mid-oogenesis, egg chambers were stained with an antibody against Dlg, a scaffolding protein that labels plasma membranes. This staining allowed for the visualization of FC membranes as they enlarged and engulfed germline material. In addition, a germline specific GFP gene trap (G89) (Morin *et al.* 2001; Meehan *et al.* 2015b) was used to visualize germline material as it was engulfed by FCs. Wild-type mid-stage death progression is shown in Figure 4, A–D, F–I.

### Quantification and statistics

Persisting nurse (PN) cell nuclei in late oogenesis were quantified in “bins” of 0, 1–3, 4–6, 7–9, 10–12, 13–15 PN per stage 14 egg chambers and presented as a percentage of total stage 14 egg chambers. The average number of PN was calculated using the median number in each bin (*e.g.*, 2 in the 1–3 bin).

Stage 13 egg chambers were scored by counting the number of nurse cell nuclei. Nurse cell nuclei that were also LysoTracker positive were scored as “acidified.” The percentage of acidified nurse cells was calculated by comparing the number of acidified NCs to the total number of NCs per stage 13 egg chamber. This method was used to quantify any acidification defects.

GraphPad Prism was used to graph and analyze all data. The nonparametric unpaired ANOVA was used for all statistical analyses.

### Data availability

The authors affirm that all data necessary for confirming the conclusions of this study are represented fully within the article and its tables and figures. All strains and reagents are available upon request. Two supplementary tables have been deposited in figshare: https://doi.org/10.25387/g3.13238942. Supplementary Table S1 is a complete list of all *Drosophila* genes identified on Flybase (Thurmond *et al.* 2019) with the GO terms relating to kinase activity. Supplementary Table S2 is a complete listing of kinase gene fly lines, their genotypes, and phenotypes used in this study.

Supplementary material is available at figshare DOI: https://doi.org/10.25387/g3.13238942.

## Results

### Identifying *Drosophila* kinase fly lines

As kinases are key regulatory enzymes involved in a variety of cellular pathways, a kinase gene RNAi screen was performed to identify novel phagocytic genes and pathways in nonprofessional phagocytes. On Flybase (Thurmond *et al.* 2019), GO terms associated with *Drosophila* genes were searched for “kinase activity” and other kinase-related terms resulting in 359 kinase genes detected in *D. melanogaster* (Supplementary Table S1). We were able to screen 224 of these genes using 328 publicly available transgenic fly lines (Supplementary Table S2).

### Identifying defects in cell corpse clearance

The GAL4-UAS system was used to identify which kinase genes played a role in the FCs, the phagocytes of the *Drosophila* ovary. The FC promoter GR1 was used to drive expression of kinase gene RNAi constructs (Figure 2A; Goentoro *et al.* 2006; Timmons *et al.* 2016). To examine how kinases affected germline death during two different stages of oogenesis, flies were reared under one of two diets: protein-conditioning (hereafter just “conditioned”) (Figure 2B) or protein-starvation (hereafter just “starved”) (Figure 2C). Once flies completed their diet regimen, their ovaries were dissected and stained with DAPI, mounted and examined for cell death and corpse clearance defects via microscopy (Figure 2, B and C). Kinase gene RNAi lines revealing phenotypes were investigated by additional RNAi fly lines when possible. Consistency among the RNAi lines is indicated in Tables 2, 4, and Supplementary Table S2.

**Table 2:** Kinase genes that demonstrate severe phenotypes during developmental death and clearance

Flybase ID	Gene name	Gene symbol	Allele	Conditioned/ developmental clearance phenotype	Additional phenotypes	Confirmed by 2nd line
**FBgn0019949**	Cyclin-dependent kinase 9	*Cdk9*	HMS01391	Very strong PN	Lose FC. NC condense, but persist.	No
**FBgn0010269**	Downstream of raf1	*Dsor1*	HMS00145	Yes	Dumpless	Yes
**FBgn0000721**	Foraging	*for*	GD6843	Strong PN	—	Yes
**FBgn0261988**	G protein-coupled receptor kinase 2	*Gprk2*	HMS00161	Strong PN	—	Yes^a^
**FBgn0027497**	MLF1-adaptor molecule	*Madm*	JF01435	Moderate PN	Dumpless.	Yes
**FBgn0250906**	Phospho-glycerate kinase	*Pgk*	HMS00030	Very strong PN	Mid-stage dying.	No
**FBgn0015277**	Phosphotidyl-inositol 3 kinase 59 F	*Pi3K59F*	HMJ30324	Very strong PN	Mid-stage death, NC nuclei degenerate.	Yes
**FBgn0015278**	Phosphotidyl-inositol 3 kinase 68 D	*Pi3K68D*	JF01193	Strong PN	—	No
**FBgn0015295**	SH2 ankyrin repeat kinase	*Shark*	JF01794	Very strong PN	—	Yes
**FBgn0016984**	Skittles	*sktl*	JF02796	Very strong PN	Dumpless, Mid-stage death	Not Tested
**FBgn0264357**	SNF4/AMP-activated protein kinase gamma subunit	*SNF4A gamma*	JF02060	Strong PN	—	No
**FBgn0045035**	Telomere fusion	*tefu*	HMS02790	Very strong PN	—	No
**FBgn0003716**	Thickveins	*tkv*	HMS04501	Strong PN	—	Yes
**FBgn0283657**	Tousled-like kinase	*Tlk*	HM05272	Strong PN	—	Not Tested
**FBgn0260935**	Vacuolar protein sorting 15	*Vps15*	HMS00908	Strong PN	Mid-stage death	Yes
**FBgn0250785**	Varicose	*vari*	HM05087	None	Undead. 15+ NC	Not Tested
**FBgn0037098**	Wnk kinase	*Wnk*	HMJ02087	Very strong PN	Dumpless	Yes

a Similar phenotype reported in a loss-of-function allele (Schneider and Spradling 1997).

**Table 4:** Kinase genes that demonstrate severe phenotypes when starved

Flybase ID	Gene name	Gene symbol	Allele	Starvation-induced mid-oogenesis phenotype	Additional phenotypes	Confirmed by 2nd line
**FBgn0014006**	Apoptotic signal-regulating kinase 1	*Ask1*	HMS00464	Engulfment defects	Undead	Not tested
**FBgn0000116**	Arginine kinase	*Argk*	JF02699	Strong engulfment defects	—	Yes
**FBgn0261854**	atypical protein kinase C	*aPKC*	HMS01411	Strong engulfment defects	—	Yes
**FBgn0015024**	Casein kinase Ialpha	*CkIalpha*	JF01792	Strong engulfment defects	—	No
**FBgn0038588**	CG7156	*CG7156*	HMJ22016	Strong engulfment defects	Undead, late phase with unfragmented NC nuclei, degenerating late-stage egg chambers, dumpless	Yes
**FBgn0030087**	CG7766	CG7766	HMJ02075	Engulfment defects	Undead	No
**FBgn0000721**	Foraging	*for*	GD6843	Strong engulfment defects	—	Yes
**FBgn0015402**	Kinase suppressor of ras	*ksr*	GL01134	Weak engulfment defects	Undead, late-stage egg chamber fragmentation	No
**FBgn0024329**	Mekk1	*Mekk1*	HM05075	Strong engulfment defects	—	Not tested
**FBgn0036187**	RIO kinase 1	*RIOK1*	HMC04524	Engulfment defects	Undead	Not tested
**FBgn0264357**	SNF4/AMP-activated protein kinase gamma subunit	*SNF4A gamma*	JF02060	Engulfment defects	Premature FC death, undead	No
**FBgn0010355**	TBP-associated factor 1	*Taf1*	HMS00416	Engulfment defects	Undead. Late NC nuclei fragmentation	Not tested
**FBgn0003716**	Thickveins	*tkv*	JF01485	Strong engulfment defects	Undead	Yes

### Screen for developmental death and clearance genes

During the final stages of oogenesis, NCs dump their intracellular contents through ring canals into the oocyte, thus providing it with the nourishment necessary for a developing embryo (Figure 3, A–D) (King 1970; Jenkins *et al.* 2013). Simultaneously, a subset of approximately 50 FCs surrounding the NC region of the egg chamber, known as stretch follicle cells (SFCs), invade the space between the nurse cells (King 1970; Spradling 1993). Once dumping is complete, the SFCs acidify the remaining NC contents which are then depleted from the egg chamber (Timmons *et al.* 2016; Mondragon *et al.* 2019; Yalonetskaya *et al.* 2020). A healthy, fully developed stage 14 egg chamber is composed of the oocyte with its newly developed chorion layer and DA surrounded by a layer of FCs (Figure 3, A and F) (King 1970; Jenkins *et al.* 2013).

Previous work has shown that the FCs are required for proper NC dumping and clearance (Timmons *et al.* 2016). In addition, it has been shown that genes associated with phagocytic pathways are required for non-autonomous developmental programmed cell death in the *Drosophila* ovary (Timmons *et al.* 2016). This can be seen when *draper* (*drpr)*, a gene encoding a key engulfment receptor (Freeman *et al.* 2003), is knocked down in FCs: NC death and clearance is incomplete and stage 14 egg chambers contain persisting NC nuclei (PN) (Figure 3G;Timmons *et al.* 2016). This form of non-cell-autonomous cell death is considered phagocyte-dependent.

To identify additional genes and pathways involved in this phagocyte-dependent developmental cell death and clearance in late oogenesis, 220 kinase genes were screened for a PN phenotype (Tables 1, 2, and Supplementary Table S2). To obtain stage 14 egg chambers, flies were conditioned by raising them on standard molasses agar plus yeast paste for 48–72 h (Figure 2B). Increased protein in their diet encourages the flies to produce more egg chambers that proceed to the final stages of oogenesis. Of the 319 lines tested, 78 lines, representing 54 genes, demonstrated a death or clearance phenotype (Tables 1, 2, and Supplementary Table S2). Of those 78 lines, two, *polo^HMS00530^* and *Tlk^HMS00943^*, were lethal when expressed with *GR1-GAL4* and were not analyzed further, and the other 76 lines survived and were found to have defective developmental death and clearance phenotypes in the ovary.

**Table 1:** Developmental death and clearance phenotype scale

Phenotype	Description	Representative gene
Very strong	75%–100% stage 14 egg chambers with >4 persisting nuclei	*tefu*
Strong	50%–75% stage 14 egg chambers with >4 persisting nuclei	*SNF4Agamma*
Moderate	25%–50% stage 14 egg chambers with >4 persisting nuclei	*rok*
Weak	10%–25% stage 14 egg chambers with >4 persisting nuclei	*Pak*
None	0%–10% stage 14 egg chambers with >4 persisting nuclei	*Adck*
Dumpless	Egg chambers where NCs do not pour their cytoplasmic contents into the oocyte	*Wnk*
Other	See comments	*Rl*
Lethal	—	*Polo*

To characterize the extent of the defects, stage 14 egg chambers were first identified by their well-defined DA and any PN were counted. Flies in which 10% or fewer of the stage 14 egg chambers contained four or more PN were characterized as having no phenotype indicated by “None” in Table 1 and Supplementary Table S2 (*e.g.*, *Adck^RNAi^*, Figure 3H, Table 1 and Supplementary Table S2). Flies in which 11–25% of the stage 14 egg chambers contained four or more PN were characterized as having a weak PN phenotype (*e.g.*, *Pak^RNAi^*, Figure 3I, Table 1 and Supplementary Table S2). Flies in which 26–50% of the stage 14 egg chambers contained four or more PN were characterized as having a moderate PN phenotype (*e.g.*, *rok^RNAi^*, Figure 3J, Table 1 and Supplementary Table S2). Flies in which 51–75% of the stage 14 egg chambers contained four or more PN were characterized as having a strong PN phenotypes (*e.g.*, *SNF4Agamma^RNAi^*, Figure 3K, Tables 1, 2, and Supplementary Table S2). Flies in which 76–100% of the stage 14 egg chambers contained four or more PN were characterized as having very strong PN phenotype (*e.g.*, *tefu^RNAi^*, Figure 3L, Tables 1, 2, and Supplementary Table S2). Of the 76 lines (51 genes) that demonstrated a PN phenotype, five lines were not further characterized, 32 lines (31 genes) demonstrated a weak PN phenotype, 20 lines (16 genes) demonstrated a moderate PN phenotype, nine lines (nine genes) demonstrated a strong PN phenotype (Table 2), and eight lines (seven genes) demonstrated a very strong PN phenotype (Table 2). In addition, six lines (four genes) demonstrated a “dumpless” phenotype in which the NC nuclei not only persisted, but also the NCs did not transfer their cytoplasmic contents into the oocyte (*e.g.*, *Wnk^RNAi^*, Figure 3M and N, Tables 1 and 2). Twenty lines (18 genes), both with and without PN, also demonstrated abnormal DA phenotypes (*e.g.*, *rl^RNAi^*, Figure 3O, Supplementary Table S2).

A number of fly lines demonstrated a phenotype during mid-oogenesis, despite being well-fed. In 22 lines (21 genes), the ovaries had an excessive number of egg chambers undergoing cell death during mid-oogenesis (*e.g.*, *Pdk1^RNAi^* and *Pi3K92E^RNAi^).* Also, in conditioned *Wnk^RNAi^* and *vari^RNAi^* flies, several egg chambers in mid-oogenesis demonstrated an “undead” phenotype in which the NCs appear healthy and uncondensed, but the FC layer is depleted (Tables 1, 2, and 4). A similar undead phenotype has been previously reported when caspases are inhibited in the germline (Laundrie *et al.* 2003; Peterson *et al.* 2003; Mazzalupo and Cooley 2006; Etchegaray *et al.* 2012). An additional 19 lines (19 genes) had other distinct phenotypes. Egg chambers where FCs expressed *CkIalpha^RNAi^* or *hop^RNAi^* did not progress beyond mid-oogenesis and those expressing *aurB^RNAi^*, *for^RNAi^*, *meng^RNAi^*, and *msn^RNAi^* had round egg chambers. *Pask^RNAi^* and *Pgk^RNAi^* led to posterior tissue accumulation, and *Pdk1^RNAi^* and *sdt^RNAi^* had egg chambers with an abnormal “bumpy” chorion (Figure 3P). Interestingly, *Pi3K59F^RNAi^*, *rl^RNAi^*, *msn^RNAi^*, *Raf^RNAi^*, *vari^RNAi^*, and *Taf1^RNAi^* demonstrated NC condensation and fragmentation instead of the acidification and disappearance typical of developmental death and clearance. This, on occasion, gave the egg chamber the appearance of having more than 15 NCs.

### Screen for starvation-induced clearance genes

Kinase genes were also screened for phenotypes during regulated cell death in mid-oogenesis. In healthy, well-fed flies, death in mid-oogenesis is sporadic, but exposure to a stressful environment, such as one lacking protein, increases the rate at which egg chambers die (Giorgi and Deri 1976; Drummond-Barbosa and Spradling 2001; Pritchett *et al.* 2009). Dying egg chambers are characterized by germline death followed by engulfment by surrounding FCs (Figure 4, A–D). To identify kinase genes required for death and clearance during mid-oogenesis, flies were starved to reduce egg chamber production and induce cell death (Figure 2C; Giorgi and Deri 1976; Drummond-Barbosa and Spradling 2001; Pritchett *et al.* 2009). The ovaries of these starved flies were then examined for defects by viewing the central plane of egg chambers in stages 7–9 using confocal microscopy. Of the 313 nonlethal fly lines tested, 75 lines representing 56 genes demonstrated an engulfment phenotype.

Dying egg chambers were characterized as either having an engulfment defect or no phenotype, indicated by “none” in Table 3 and Supplementary Table S2. Engulfment defective phenotypes were further characterized as having a weak or strong phenotype (Tables 3, 4, and Supplementary Table S2). When genes that regulate clearance of the germline are knocked down in the FCs, the FC layer has been shown to die prematurely, leaving unengulfed germline (Etchegaray *et al.* 2012). Thus, when phagocytic genes, such as *drpr*, *Rac1*, and *shark*, are perturbed in the FCs, the germline continues to undergo apoptosis, but the FCs die before engulfing the germline (Figure 4E), which is distinct from the wild-type engulfment process where FCs persist until engulfment of the germline is complete (Figure 4, F–I). Thus, a dying egg chamber with an intact and engulfing FC layer was characterized as having no phenotype (*e.g.*, *CG10702^RNAi^*, Figure 4J, Table 3 and Supplementary Table S2). A dying egg chamber with pyknotic and/or missing FCs was characterized as having a weak engulfment defect (*e.g.*, *LIMK1^RNAi^*, Figure 4K, Table 3 and Supplementary Table S2). Dying egg chambers that were missing most or all of their FCs and unengulfed germline, similar to *drpr* mutants (Figure 4E), were characterized as having strong engulfment defects (*e.g.*, *aPKC^RNAi^*, Figure 4L, Tables 3, 4, and Supplementary Table S2) (Meehan *et al.* 2015a). In addition, several fly lines also produced egg chambers that could be characterized as “undead”—in which the germline had yet to commence apoptosis, but the FC layer had already been depleted (*e.g.*, *Taf1^RNAi^*, Figure 4M, Tables 3, 4, and Supplementary Table S2) (Laundrie *et al.* 2003; Peterson *et al.* 2003; Mazzalupo and Cooley 2006; Etchegaray *et al.* 2012).

**Table 3:** Mid-oogenesis death and engulfment scale

Phenotype	Description	Representative gene
Strong	Egg chamber with condensing and fragmenting chromatin missing most, if not all, FCs.	*aPKC*
Weak	Egg chamber with condensing and fragmenting chromatin missing some FCs.	*LIMK1*
None	Egg chamber with a complete, living layer of FCs	*CG10702*
Undead	Egg chamber with dispersed, healthy-looking chromatin missing most, if not all, FCs.	*Taf1*
Other	See comments	*Cdk1*
Lethal		*polo*

Of the 75 lines (56 genes) that demonstrated an engulfment-related phenotype, 50 lines (40 genes) demonstrated a weak phenotype, seven lines (seven genes) demonstrated a strong phenotype (Table 4), and, in addition to the two lines (genes) from conditioned diets, nine lines (eight genes) demonstrated an undead phenotype when starved (Table 4). In addition, 24 lines (22 genes) demonstrated distinct phenotypes beyond engulfment including *Cdk1^RNAi^* which had few mid-stage egg chambers and severe defects in stage 6 egg chambers; *hop^RNAi^* in which egg chambers did not progress past stage 6 or 7; and *meng^RNAi^*, *pelle^RNAi^*, *msn^RNAi^*, and *aurB^RNAi^* which all demonstrated misshapen egg chambers.

### *Pi3K59F* and *Pi3K68D* are required for nurse cell acidification and clearance

**Figure 5:**
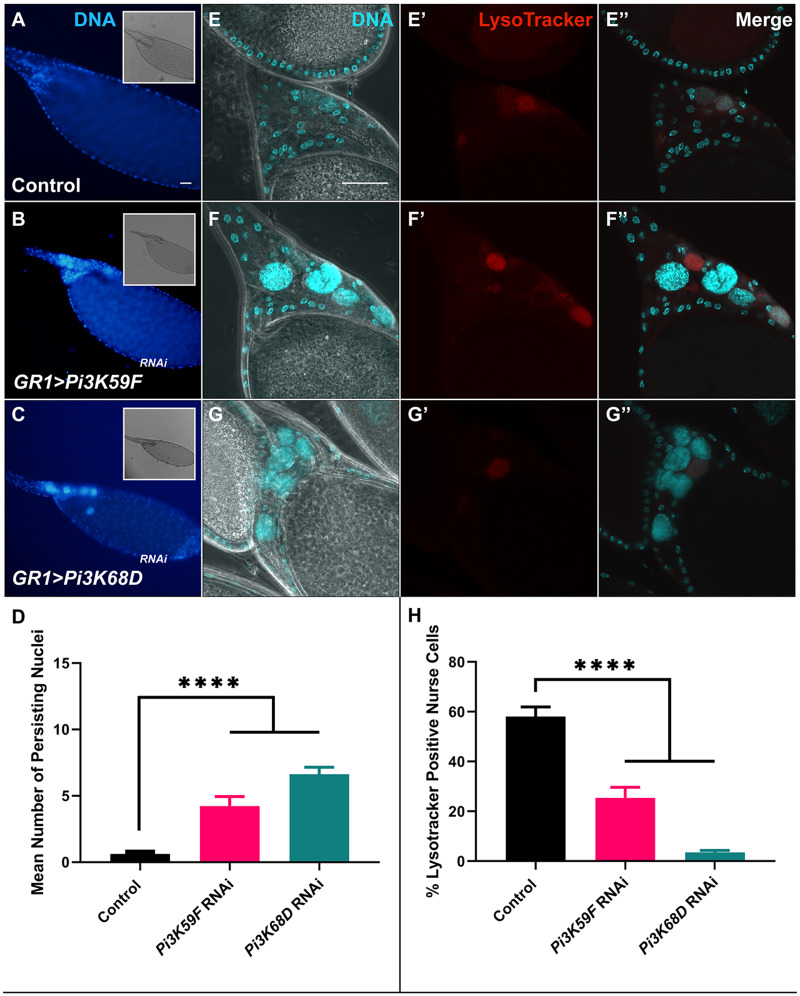
Knockdowns of classes II and III PI3-kinases result in strong defects during late oogenesis. (A–C) Stage 14 egg chambers from well-fed flies stained with DAPI (blue) to label DNA. Insets—Egg chambers imaged with DIC to identify stage 14 egg chambers using DAs. (A) Sibling control egg chambers did not have PN. (B) *GR1>Pi3K68D^JF0119^* egg chambers exhibited a strong PN phenotype. (C) *GR1>Pi3K59F^HMS00261^* egg chambers exhibited a strong PN phenotype. (D) Quantification of PN in sibling control, *GR1>Pi3K59F^HMS00261^*, and *GR1>Pi3K68D^JF0119^* stage 14 egg chambers. Data presented are mean + SEM. (E–G) Stage 13 egg chambers from well-fed flies stained with DAPI (cyan) to label DNA. DIC was used to visualize the developing DA to identify egg chambers as stage 13. (Eʹ–Gʹ) Egg chambers labeled with LysoTracker (red) to identify acidified compartments. (Eʹʹ–Gʹʹ) Egg chambers co-labeled with DAPI (cyan) and LysoTracker (red). (E–Eʹʹ) Sibling control egg chambers contained a few acidified NCs, with the majority already degraded. (F–Fʹʹ) *GR1>Pi3K68D^JF0119^* contained several NC nuclei that were not LysoTracker positive. (G–Gʹʹ) *GR1>Pi3K59F^HMS00261^* contained several nuclei, several of which were not LysoTracker positive. (H) Quantification of LysoTracker staining in stage 13 egg chambers. Data presented are mean + SEM. All scale bars depict 50 μm. Scale bar in (E) is representative of images in (E–Gʹʹ).

One class of kinases that emerged from the screen were the three PI3 Kinases, which were followed up in more detail. Pi3K59F is the *Drosophila* class III phosphoinositide 3-kinase, otherwise known as Vps34. Vps34 was first found in yeast and is known to play a role in autophagy (MacDougall *et al.* 1995; Vanhaesebroeck *et al.* 1997; Wymann and Pirola 1998; Backer 2008; Nezis *et al.* 2010; Jean and Kiger 2014) and phagocytosis in *C. elegans* and mammals (Lu *et al.* 2012; Heckmann *et al.* 2017). *Pi3K59F^RNAi^* produced a strong PN phenotype where stage 14 egg chambers contained an average of four PN per stage 14 egg chamber (Figure 5, A, B, and D). The NCs were able to successfully dump their cytoplasmic contents into the oocyte as was evident by the lack of cytoplasmic material surrounding the NC nuclei; only the DNA remained (Figure 5B).

As wild-type developmental nurse cell death progresses, NC nuclei are acidified during stages 12 and 13 (Bass *et al.* 2009; Mondragon *et al.* 2019). To determine if *Pi3K59F* affected NC acidification, *Pi3K59F^RNAi^* flies were stained with LysoTracker. Some stage 13 egg chambers with NC nuclei were LysoTracker positive, indicating that some of the NCs were being acidified (Figure 5, E–Fʹʹ). However, the percentage of acidified NCs was significantly decreased compared to sibling controls (Figure 5H). *Pi3K59F^RNAi^* stage 13 egg chambers had 26% acidified nuclei compared to 58% acidified nuclei in control flies (Figure 5H). These findings suggest that the acidification process was disrupted or delayed.

Vps34 complexes with Vps15 to form a heterodimer, that is required for autophagy and endocytosis (MacDougall *et al.* 1995; Vanhaesebroeck *et al.* 1997; Wymann and Pirola 1998; Backer 2008; Jean and Kiger 2014). Consistent with this, we found that *Vps15^RNAi^* produced a moderate or strong PN phenotype (Supplementary Table S2). These data suggest that *Pi3K59F* or its substrates may be required in the FCs in late-stage oogenesis to regulate NC acidification and elimination.

Pi3K68D is the *Drosophila* class II phosphoinositide 3-kinase. While class II PI3K function has been characterized in humans, its role and function are still relatively unknown in *Drosophila* (MacDougall *et al.* 1995; Vanhaesebroeck *et al.* 1997; Wymann and Pirola 1998; Jean and Kiger 2014). *Pi3K68D^RNAi^* flies also demonstrated decreased NC acidification and a strong PN phenotype (Figure 5, C–H). Stage 14 egg chambers had an average of 6 PN and only 4% of stage 13 NC nuclei became acidified, indicating a role for *Pi3K68D* in FCs to facilitate NC acidification and elimination.

### *Pi3K92E* is required for the progression of engulfment during starvation-induced death

**Figure 6:**
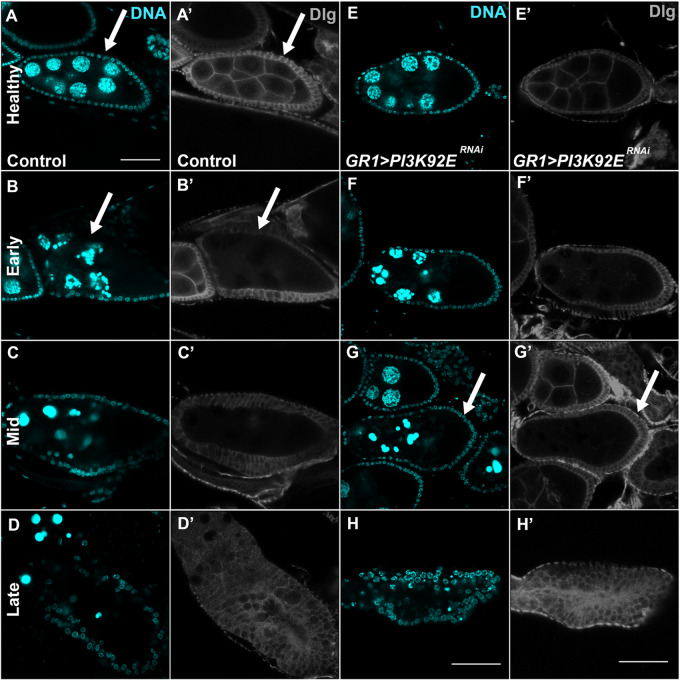
Knocking down of class I PI3K results in delayed engulfment during mid-oogenesis. (A–C) Sibling control egg chambers stained with DAPI (cyan) to visualize the nuclei of healthy, early dying, and mid phase dying respectively. (D) *GR1>Luc^RNAi^* egg chamber stained with DAPI (cyan) to visualize nuclei. (Aʹ–Dʹ) Control egg chambers stained with α-Dlg (grey) to label membranes of FCs and NCs. (A–Aʹ) Healthy egg chamber shows a thin FC layer surrounding germline NCs. (B–Bʹ) Egg chamber undergoing early phase of death shows a thin FC layer surrounding condensing and fragmenting NC nuclei. (C–Cʹ) Egg chamber in mid-phase death continues to show condensing NC nuclei while the FC layer increases in size as it begins to engulf germline material. (D–Dʹ) Late phase dying egg chamber shows a FC layer that completely encroaches on the germline region as it engulfs any remaining condensed NC nuclei and other germline material. (E–Hʹ) *GR1>Pi3K92E^RNAi^* (*Pi3K92E^JF02770^*) egg chambers in various phases of health and death. (E–H) *GR1>Pi3K92E^RNAi^* egg chambers stained with DAPI (cyan) to visualize the nuclei of healthy, early dying, mid, and late phase dying respectively. (Eʹ**–**Hʹ) *GR1>Pi3K92E^RNAi^* egg chambers stained with α-Dlg (grey) to label membranes of FCs and NCs. (E–Eʹ) Healthy egg chambers demonstrating diffuse NC nuclei and a single thin layer of FCs. (F–Fʹ) Early phase dying *GR1>Pi3K92E^RNAi^* egg chamber with condensing nuclei with no change in the FC layer. (G–Gʹ) Mid phase dying *GR1>Pi3K92E^RNAi^* egg chamber with nuclei undergoing further condensation and fragmentation, but the FC layer still has not enlarged to start clearing the germline as it has in the control. (H–Hʹ) Late phase dying *GR1>Pi3K92E^RNAi^* egg chamber where the germline has been cleared by an enlarged FC layer. All scale bars depict 50 μm. Scale bar in A is representative of all images in this figure unless otherwise indicated.

Pi3K92E is the *Drosophila* class I phosphoinositide 3-kinase. Pi3K92E has been well-characterized in *Drosophila* and other organisms as part of the PI3K/AKT/TOR pathway (MacDougall *et al.* 1995; Vanhaesebroeck *et al.* 1997; Wymann and Pirola 1998; Jean and Kiger 2014). Knocking down *Pi3K92E* in FCs did not produce a strong engulfment defect phenotype in mid or late-stage oogenesis. However, there was a partial delay in engulfment in mid-oogenesis in starved flies. Previously, we described phases of cell death in mid-oogenesis where the state of nurse cell chromatin changes is correlated with enlargement of and engulfment by FCs (Etchegaray *et al.* 2012). Figure 6 shows these phases of death in a mid-stage egg chamber in control and *Pi3K92E^RNAi^* flies. Healthy (not dying) egg chambers showed dispersed NC chromatin surrounded by an organized layer of FCs (Figure 6, A–Aʹ, E–Eʹ). As egg chambers began to die, nurse cell chromatin condensation was observed but the FC layer remained thin and the germline was not engulfed (Figure 6, B–Bʹ). *Pi3K92E^RNAi^* early dying egg chambers resembled those of sibling controls (Figure 6, F–Fʹ).

Mid-dying egg chambers are characterized by NC chromatin condensation with apparent individual nuclear regions, and the FCs enlarge and engulf the dying germline. As egg chambers progress, NC chromatin becomes highly condensed into balls of DNA. In wild-type egg chambers, the FC membrane enlarges to about triple its original thickness, and the germline region becomes visibly smaller as it is engulfed by FCs (Figure 6, C–Cʹ). Many *Pi3K92E^RNAi^* egg chambers exhibited a delay in FC membrane enlargement during mid-phases of death. Egg chambers characterized as mid-phase based on chromatin morphology (Figure 6, G–Gʹ) had FC membranes that resembled early phase dying egg chambers (Figure 6, Bʹ, Fʹ). In addition, there was no obvious reduction of the germline region, suggesting there was little engulfment of the germline material. As death progressed, *Pi3K92E^RNAi^* FCs did eventually enlarge to resemble wild-type late phase dying egg chambers (Figure 6, D–Dʹ, H–Hʹ), suggesting a delay in engulfment progression.

### *Pi3K92E* and *Pdk1* knockdowns cause excessive mid-stage cell death in well-fed flies

**Figure 7:**
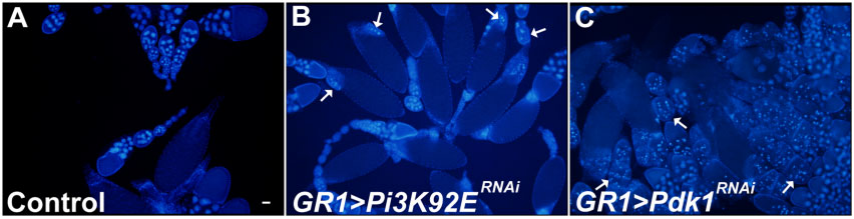
Members of the PI3K/PTEN/AKT pathway are required for egg chamber survival during mid-oogenesis in well-fed flies. (A**–**C) Low magnification images displaying multiple egg chambers labeled with DAPI (blue). White arrows indicate degenerating egg chambers. (A) *GR1>Luc^RNAi^* egg chambers from well-fed conditioned flies. No degenerating egg chambers are observed. (B) *GR1>Pi3K92E^JF02770^* egg chambers from well-fed (conditioned) flies demonstrate a moderate level of degeneration during mid-oogenesis. (C) *GR1>Pdk1^JF02807^* egg chambers from well-fed (conditioned) flies demonstrated an excessive amount of degeneration during mid-oogenesis.

Interestingly, *Pi3K92E^RNAi^* flies also produced mid-stage dying egg chambers when they were well-fed (conditioned) (Figure 7B). *Pdk1^RNAi^*, another member of the PI3K/AKT/TOR pathway (Cho *et al.* 2001)^,^ also demonstrated mid-stage degenerating egg chambers when conditioned (Figure 7C). Based on nuclear condensation and fragmentation, these egg chambers would be characterized as mid-phase dying egg chambers. However, these egg chambers contained a large amount of unengulfed germline material and the FCs failed to enlarge. Interestingly, flies from either genotype had normal numbers of degenerating egg chambers under nutrient deprivation, suggesting that *Pi3K92E/PDK1* protect against degeneration only when well-fed.

## Discussion

Here, we have reported a kinome screen of FCs, with a focus on engulfment and clearance. Of the 224 genes (328 fly lines) tested, 93 genes (140 lines) demonstrated a phenotype. Most genes fell within six phenotypic categories—engulfment defective, persisting nuclei, both engulfment defective and persisting nuclei, undead, dumpless, or excessive mid-oogenesis death when conditioned (Table 5). Here, these genes will be further discussed based on the category of phenotype they displayed during the screen. The diverse effects of the PI3Ks will also be addressed.

**Table 5:** Kinase RNAi phenotype categories

Category of kinase phenotype	Gene symbol(s)
Both engulfment and developmental death and clearance	*Ack, AdenoK, aPKC, Ask1, bsk, Cdk1, Cdk2, Cdk9, CG7766, CkIalpha, CycT, Dgk, Dsor1, for, Gprk2, mei-41, Pak, Pi3K59F, Pi3K68D, pll, polo, Rok, S6k, Shark, sktl, SNF4Agamma, Taf1, Tlk, vari, Vps15*
Developmental death and clearance	*Ack-like, aurB, CG11486, CG2577, Dyrk3, fj grp, JIL-1, Lk6, Ikb1, Madm, men, msn, Pask, Pdk1, Pgk, Raf, rl, sdt, sti, Stlk, Tao, tefu, Wnk*
Engulfment during starvation	*Argk, Atg1, bt, CaMKI, CASK, Cdc7, Cdk5alpha, CG10082, CG10177, CG3007, CG7156, dlg1, Eph, GckIII, gish, hop, hpo, LIMK1, Mekk1, nmo, Pi3K92E, Pvr, RIOK1, Sdr, Slik, slpr, trc, vari*
Nurse cell dumping	*Dsor1, Madm, sktl, Wnk*
Nurse cell death and FC survival during mid-oogenesis	*Ask1, CG7766, RIOK1, SNF4Aγ, Taf1, vari*
Required for cell survival during mid-oogenesis	*Ask1, CkIalpha, CG10082, msn, niki, Pgk, Pdk1, Pi3K92E, Pkc92E, PKD, Pkn, put, Raf, Sdr, sktl, tkv, Vps15, wit*

### Kinase genes required for both engulfment and developmental death and clearance

Twenty-seven kinase genes demonstrated a phenotype during both starvation-induced and developmental death and clearance (Table 5). Although the processes of developmental and starvation-induced death and clearance differ there are many congruencies as well. For instance, the germline/NCs receive some signal to die, the FCs change shape to surround the germline/NCs, and the germline/NCs are depleted. Moreover, phagocytosis machinery and the JNK pathway are required during both processes (Etchegaray *et al.* 2012; Timmons *et al.* 2016). While it is possible that these kinases have different functions depending on the form of death, it is also possible that these genes may play similar roles during both death events.

Some of the strongest phenotypes in this group were demonstrated by *Shark*, *Gprk2*, *Cdk9*, and *for* (Tables 2 and 4). *Shark* encodes a kinase that interacts with the cytosolic region of Drpr. Previous studies have shown that without *Shark*, engulfment during starvation-induced death is defective and nuclei are not fully cleared during developmental death and clearance (Etchegaray *et al.* 2012; Timmons *et al.* 2016).

*Gprk2* is a protein originally found to regulate GPCRs through phosphorylation (Cassill *et al.* 1991; Schneider and Spradling 1997). A recent study in mice found that an ortholog of *Gprk2*, *GRK6*, is required for proper clearance of apoptotic cells. Interestingly, GRK6 did not interact with the two known engulfment pathways in mammals (Nakaya *et al.* 2013).

The kinase For plays a role in several processes including feeding behavior and response to stimulus and has effects on metabolism and the insulin pathway (Pereira and Sokolowski 1993; Belay *et al.* 2007; Kent *et al.* 2009). *for* has also been found to interact with *lola* (Peng *et al.* 2016), a gene previously shown to be required for programmed cell death in the *Drosophila* ovary (Bass *et al.* 2007). Thus, *for* may regulate clearance via metabolism, the insulin pathway or its interaction with *lola*.

*Cdk9* encodes a transcription elongation factor (Marshall and Price 1995). As a regulator of transcription, it has effects on multiple processes including the response to heat and viruses (Lis *et al.* 2000; Panda *et al.* 2014). *Cdk9* and *for* demonstrate that some of the genes identified may not only be necessary for proper clearance, but overall cell function and survival.

### Kinase genes required for developmental death and clearance

During developmental death and clearance, the NCs dump their cytoplasmic contents through ring canals into the oocyte. As dumping proceeds, the stretch FCs invade the space between the NCs. Once dumping is complete, the stretch FCs acidify the nurse cell nuclei, leading to their clearance (Timmons *et al.* 2016; Mondragon *et al.* 2019). Kinase genes that play a role in developmental death and clearance may regulate the invasion of the stretch FCs between the NC. However, they may also have a direct effect on removal of the NCs. For instance, V-ATPases of the FCs regulate the acidification of the NCs (Mondragon *et al.* 2019). Kinase genes may also affect the acidification of the nurse cells or there may be other signals being exchanged.

Developmental death and clearance were specifically perturbed by 35 kinase genes. The most common defect displayed was persisting nurse cell nuclei in stage 14 egg chambers. The strongest PN phenotypes were displayed by *Pgk^RNAi^* and *tefu^RNAi^* (Table 2).

*Pgk* encodes phosphoglycerate kinase which plays a role in muscle development and acts as a genetic enhancer of *Dystroglycan*, a protein that transmits information from the extracellular matrix to the cytoskeleton (Kucherenko *et al.* 2008). *Pgk* may therefore regulate the cytoskeletal changes that take place in stretch FCs as they interact with and clear nurse cells.

*Tefu* produces a serine-threonine kinase responsible for maintaining genome integrity in cells participating in the cell cycle and DNA repair (Savitsky *et al.* 1995). Mutating the kinase region of *tefu* has been shown to cause neurodegeneration in the brain through an uncontrolled inflammatory response (Petersen *et al.* 2012). *Tefu*, like *Cdk9* and *for*, shows how integral genes are required for the proper survival and function of NCs and FCs.

### Kinase genes required for engulfment during starvation

During starvation-induced death, the germline undergoes a predominantly apoptotic death while the FC layer enlarges and engulfs the germline (Peterson *et al.* 2003; Mazzalupo and Cooley 2006; Pritchett *et al.* 2009; Serizier and McCall 2017). Kinase genes that affected death during mid-oogenesis may regulate aspects of the synchronous enlargement and engulfment by the FCs. In addition, as the FCs and germline are intimately associated, these kinase genes may lead to signals that affect the ability of the FCs to recognize the germline as dying.

Starvation-induced clearance was perturbed by the knocking down of 56 kinase genes. Most of these genes demonstrated engulfment defects where the FC layer died before engulfing the germline, similar to previously described *drpr* mutants (Etchegaray *et al.* 2012). The strongest defects were exhibited by *aPKC^RNAi^*, *Argk^RNAi^*, and *Mekk1^RNAi^* (Table 4). aPKC is a component of the Par complex which establishes cell polarity. aPKC phosphorylates proteins such as Baz to exclude them from the apical domain (Morais-de-Sá *et al.* 2010). In a previous study, it was demonstrated that the aPKC-dependent polarization of the FCs was required for proper engulfment during starvation-induced death (Meehan *et al.* 2015a).

Little is known about the function of *Argk*. Much of what we know comes from its mammalian ortholog, *creatine kinase* (*CK*), which plays a role in energy homeostasis by regulating phosphate transfer from ATP during intermittent high-energy demands. During a previous study, it was found that loss of *CK* expression resulted in the loss of energy and subsequent loss of actin remodeling at the phagocytic cup of macrophages, thus precluding engulfment (Kuiper *et al.* 2008).

*Mekk1* encodes a MAPKKK that is required to resist oxidative and other environmental stresses (Inoue *et al.* 2001). In a previous study, it was found that Mekk1, an upstream activator of the JNK pathway, was required for FC enlargement, engulfment, and survival during starvation-induced death (Etchegaray *et al.* 2012). Additional JNK family components that demonstrated an engulfment phenotype were *Ask1*, *slpr*, and *bsk* (Etchegaray *et al.* 2012).

### Kinase genes required in the FCs for nurse cell dumping

During stage 11, the NCs dump their cytoplasmic contents through ring canals into the oocyte (Timmons *et al.* 2016). This dumping is essential for oocyte growth and development into an embryo (Gutzeit 1986). Several studies have linked proper dumping to actin regulators such as *chic* (Cooley *et al.* 1992), *sn* (Cant *et al.* 1994), *cpb* (Ogienko *et al.* 2013), and *qua* (Mahajan-Miklos and Cooley 1994) within the NC. In a previous study, we demonstrated that the FCs were required for the dumping of cytoplasmic contents and subsequent nurse cell death. When stretch FCs were ablated by expressing *Diap1^RNAi^*, most of the NCs persisted and retained their cytoplasm (Timmons *et al.* 2016). In this study, four kinases demonstrated a dumpless phenotype when knocked down in FCs—*Dsor1*, *Madm*, *sktl*, and *Wnk* (Table 2).

Wnk is a chloride-sensitive serine/threonine kinase that is involved with both Wnt signaling as well as ion transport (McCormick and Ellison 2011; Serysheva *et al.* 2013)^.^ Interestingly, in a previous study it was determined that the chloride channel, ClC-b, was enriched on the membrane of stretch FCs during developmental death and clearance (Mondragon *et al.* 2019). Thus, ClC-b and Wnk may interact with one another to regulate the dumping process. As of yet, the four dumpless genes have not been associated with one another. This suggests that either there are four separate pathways that control dumping or that there is an undiscovered connection between some or all of these genes.

### Kinase genes required for nurse cell death and FC survival during mid-oogenesis

The undead phenotype is a previously described two-part phenotype that affects both the NCs and the FCs (Laundrie *et al.* 2003; Peterson *et al.* 2003; Mazzalupo and Cooley 2006; Etchegaray *et al.* 2012). In undead egg chambers, the NC nuclei fail to condense and fragment in the wake of protein-starvation. Instead, the FC layer disappears leaving the germline behind (Laundrie *et al.* 2003; Peterson *et al.* 2003; Etchegaray *et al.* 2012). Previous studies have uncovered genes that can induce the undead phenotype, including *Diap1* and *Dcp-1*. When *Diap1* is overexpressed in the germline, caspases, such as *Dcp-1*, remain inactive, thus leaving the NCs intact (Peterson *et al.* 2003). Null mutations in *Dcp-1*, the primary effector caspase of starvation-induced death, have a similar effect (Laundrie *et al.* 2003; Etchegaray *et al.* 2012; Pritchett and McCall 2012). In this study, we identified 10 kinase genes that, when knocked down in the FCs, also demonstrated the undead phenotype—*Taf1*, *ksr*, *Wnk*, *vari*, *CG7766*, *CG7156*, *Ask1*, *tkv*, *RIOK1*, and *SNF4Agamma* (Tables 2 and 4).

*CG7766* is an uncharacterized gene that has been predicted by Flybase.org to be involved in calmodulin binding and act as phosphorylase kinase. BLAST analysis (Altschul *et al.* 1990) of the *CG7766* coding region recapitulated the phosphorylase b kinase prediction. *Ask1* transmits stress responses thus inducing cell death and immunity (Ichijo *et al.* 1997; Hayakawa *et al.* 2012). *RIOK1* has also been described as an atypical protein kinase. During another kinome RNAi screen in the brain, it was determined that *RIOK1* and *RIOK2* modulate Akt signaling (Read *et al.* 2013). *Ask1* is a MAPKKK that signals in both the JNK and p38 pathways (Wassarman *et al.* 1996). *SNF4Agamma* encodes the regulatory subunit of AMPK and plays a role in several functions including the starvation response and autophagy (Lippai *et al.* 2008; Johnson *et al.* 2010). Unlike the dumpless phenotype, some of these 10 kinases have been connected to one another. *SNF4Agamma* and *RIOK1* have been shown to play a role in TOR signaling (Johnson *et al.* 2010; Read *et al.* 2013), a hub of nutrient and environmental sensing. As such, Tor plays a key role in regulating the development of egg chambers through mid-oogenesis (Loewith and Hall 2011; Pritchett and McCall 2012; Lushchak *et al.* 2017). In addition Ask1, RIOK1 and SNF4Agamma have all been connected to cell death pathways (Ichijo *et al.* 1997; Lippai *et al.* 2008; Hayakawa *et al.* 2012; Read *et al.* 2013).

Prior to this study, it was thought that genes expressed in the germline were the sole cause of the undead phenotype while FCs were simply bystanders, dying and being cleared because of inadequate signaling or nutrients from NCs. The effects of these FC-specific knockdowns suggest that genes expressed in the FCs can also play a role in affecting the undead phenotype. While it is clear that the kinases must lead to some death signal(s) for the germline, the question remains as to whether the kinases also regulate the survival of and engulfment by the FCs. Do the FCs fail to survive and engulf because the germline is not receiving the signal to die or knocking down the kinase simply make the FCs sensitive to starvation?

### PI3Ks differentially regulate clearance pathways

PI3Ks phosphorylate the hydroxyl group of position 3 on the inositol ring of phosphoinositides to induce different signals. They have been implicated in regulating proliferation, survival, metabolism, cytoskeletal rearrangements, and membrane trafficking. PI3K genes have been divided into three classes based on their structure and function, with one representative of each found in *Drosophila—Pi3K92E*, *Pi3K68D*, and *Pi3K59F* (MacDougall *et al.* 1995; Vanhaesebroeck *et al.* 1997; Wymann and Pirola 1998; Jean and Kiger 2014).

In this study, *Pi3K68D* and *Pi3K59F* were both found to affect acidification and clearance in late oogenesis. A previous study in *C. elegans* showed that class II and class III PI3Ks act sequentially to promote two waves of Phosphatidylinositol 3-P [PI(3)P] generation for proper phagosome maturation. A first wave of membrane PI(3)P generation by class II PI3K may be required to initiate phagosome maturation through specific downstream effectors. Class III PI3K is required to maintain proper levels of phagosomal PI(3)P and regulate the second wave of PI(3)P generation to complete phagosome maturation. Phosphatase Mtm also works to regulate PI(3)P turnover between the two waves. This PI(3)P turnover may allow for recruitment of different downstream effectors during each wave of PI(3)P generation to promote different stage of phagosome maturation (Lu *et al.* 2012). These findings suggest that PI3Ks may be regulating nurse cell acidification and clearance in a stepwise manner similar to *C. elegans*.

While *Pi3K92E* was not required for clearance in mid or late-stage oogenesis, the delayed engulfment phenotype exhibited during mid-oogenesis suggests that Pi3K92E signaling could temporally affect engulfment progression. Previous studies have shown that Pi3K92E signaling modulates Drpr levels in the *Drosophila* brain (Doherty *et al.* 2014). In the brain, glial cells have a phagocytic function via Drpr in response to axonal injury. Decreased *Pi3K92E* leads to a decrease in glial Drpr, but does not completely deplete glial Drpr levels or inhibit axon debris clearance. Instead, axon clearance is delayed, but ultimately all of the debris is cleared.

This evidence suggests a link between Pi3K92E, Drpr, JNK, and engulfment in the *Drosophila* ovary. Previous work has shown that Drpr is required in FCs for engulfment during mid-stage oogenesis and JNK signaling acts downstream of Drpr to promote engulfment (Etchegaray *et al.* 2012). *Pi3K92E^RNAi^* flies showed a delay in FC engulfment similar to the engulfment delays in the *Drosophila* brain, thus *Pi3K92E* may be required in FCs to regulate basal Drpr levels to initiate FC engulfment. Once Drpr is activated, it may signal through JNK to promote enrichment of Drpr and other engulfment genes to stimulate germline clearance.

### Kinase genes of the PI3K/PTEN/AKT pathway may be required for cell survival

During this study, 22 genes, when knocked down in the FCs, induced excessive egg chamber death during mid-stage oogenesis despite being well-fed. The excessive degenerating phenotype from *Pi3K92E^RNAi^*and *Pdk1^RNAi^* under nutrient rich conditions suggests a link between the insulin signaling pathway and induction of cell death. Previous work has shown that insulin signaling and the TOR pathway are required in FCs to transmit nutrient information to the germline to mount a starvation response (Burn *et al.* 2015).

How insulin signaling induces cell death in the germline is still unknown? Insulin signaling is directly involved in cell growth and survival, but there is no direct link between insulin signaling and cell death. Three other related genes also produced an excessive mid-oogenesis death under well-fed conditions, *Sdr, Raf*, and *Pkc53E* (Carnero and Paramio 2014; Hemmings and Restuccia 2015; Alfa and Kim 2016). While *Sdr* is also in the insulin signaling pathway (Alfa and Kim 2016), the other two are related via the more central PI3K/PTEN/AKT signaling pathway (Carnero and Paramio 2014; Hemmings and Restuccia 2015). While the one *Akt* RNAi fly line tested during our investigation showed no phenotype, previous research has demonstrated that *Akt* regulates the size and shape of FCs as well as their migration. In addition, overexpression of *Akt* resulted in a dumpless phenotype and an excess number of nuclei within the oocyte (Cavaliere *et al.* 2005). Therefore, kinases and other genes associated with Akt signaling are ripe for further investigation in the FCs.

### Concluding remarks

During this study, 224 kinase genes were examined to identify phenotypes during the starvation-induced and developmental death and clearance events of oogenesis. Of these, 93 unique genes demonstrated a phenotype during some form of death and clearance. As proof of concept, several of these genes were previously identified including *Shark*, *Mekk1*, and *aPKC* (Etchegaray *et al.* 2012; Meehan *et al.* 2015a).

While previous studies guided our search toward egg chambers with either defective engulfment or persisting nuclei, our unbiased screen allowed us to find 15 additional kinase genes that exhibited unique phenotypes such as delayed engulfment and excessive death during mid-oogenesis in well-fed flies. These newly identified genes, including *Pi3K92E* and *PDK1*, may provide a new avenue of research. In addition to these unique phenotypes, we also identified several instances of lines producing dumpless and undead phenotypes. Exploration of dumpless genes acting in the FCs may allow us to parse out the early stages of developmental death. Most descriptions of the undead phenotype have been attributed to genes required in the germline, yet we have demonstrated a requirement in the FCs. Finding undead genes among the FC expressed kinases was particularly surprising. These undead kinase genes provide evidence that germline death during mid-oogenesis is controlled non-cell-autonomously and creates yet another direction of research.

The major limitation of this study is its dependency on RNAi technology. We used RNAi fly lines available at the start of this study and frequently acquired newly developed stocks, but due to reagent limitations, not every kinase has been thoroughly studied in our screen. Further study may identify additional kinases that affect death and clearance during oogenesis. In addition, several genes that should have exhibited a phenotype, such as *Akt*, did not. This may be due to inefficient RNAi targeting. With the development of transgenic lines carrying gene-specific guideRNAs, CRISPR/Cas9 can be used to further investigate the requirement for kinase genes in FCs.

Future analysis will determine if the kinases act in one of the previously identified pathways (*e.g.*, Drpr, Ced-12, JNK or cell polarity) (Ellis *et al.* 1991; Franc 2002; Kinchen *et al.* 2005; Mangahas and Zhou 2005; Meehan *et al.* 2015a; Hursh *et al.* 2016; Zheng *et al.* 2017) or if there is a heretofore unknown pathway waiting to be discovered. Additional tests in other phagocytic cells, such as glia and hemocytes, can determine whether the clearance effects of these kinases are ubiquitous or tissue-specific, as well as pan-phagocytic or specific to nonprofessional phagocytes.
